# Impact of adjuvant radiation therapy after definitive surgery in senior adults >80 years old with advanced head and neck squamous cell carcinoma on overall survival

**DOI:** 10.3389/fonc.2022.973245

**Published:** 2022-09-30

**Authors:** Joann M. Butkus, Meghan Crippen, Voichita Bar-Ad, Adam Luginbuhl

**Affiliations:** ^1^ Department of Otolaryngology- Head & Neck Surgery, Thomas Jefferson University, Philadelphia, PA, United States; ^2^ Department of Radiation Oncology, Thomas Jefferson University, Philadelphia, PA, United States

**Keywords:** senior adult, adjuvant radiotherapy, head and neck cancer, survival, National Cancer Database

## Abstract

**Background:**

Adjuvant radiotherapy (RT) following surgical resection confers a survival benefit for adult patients with locally advanced head and neck squamous cell carcinoma (HNSCC). We aim to investigate if adjuvant RT provides a similar survival advantage to patients ages 80+ through a national curated database.

**Methods:**

This retrospective cohort study queried the National Cancer Database (NCDB) for all cases of HNSCC between 2004-2016. Patients treated with surgical resection alone were compared to those treated with surgery plus adjuvant RT. Overall survival (OS) was compared within adult (age <80 years) and senior adult (age ≥80 years) cohorts using Kaplan-Meier analysis. Hazard ratios (HR) were assessed using Cox proportional hazards to account for differences in patient characteristics, primary site, and HNSCC stage.

**Results:**

NCDB identified 16,504 locally advanced HNSCC treated with definitive surgery with 9,129 (55.3%) also receiving adjuvant RT. The mean age was 63.8 years (SD = 12.0) with 88.7% of patients ages <80 years and 11.3% ages ≥80 years. In the adult cohort, adjuvant RT was associated with a significant increase in OS compared to surgery alone at 1 year (88.4% vs. 83.8%, p=<0.001), 3 years (64.0% vs. 59.2%, p=<0.001) and 5 years (52.8% vs. 47.2%, p=<0.001). Treatment with surgery alone remained a significant predictor of mortality risk at 1 year (HR 1.48, 95% CI 1.35-1.64, p<0.001), 3 years (HR 1.25, 95% CI 1.18-1.33, p<0.001), and 5 years (HR of 1.23, 95% CI 1.17-1.30, p=<0.001). In the senior adult cohort, there were no significant differences in OS between treatment groups at 1 year (73.4% vs. 74.8%, 0.296), 3 years (45.8% vs. 41.8%, p=0.465), or 5 years (28.2% vs. 27.7% p=0.759). Treatment with surgery alone was not a significant predictor of mortality risk at 1 year (HR 1.11, 95% CI 0.90-1.36, p=0.316), 3 years (HR 0.94, 95% CI 0.81-1.08, p=0.423), or 5 years (HR 0.95, 95% CI 0.83-1.08, p=0.476).

**Conclusion:**

The addition of adjuvant RT in senior patients (age ≥80 years) may not provide a similar OS benefit to that observed in younger patients. Further research is needed to best guide shared-decision making in this population.

## Introduction

According to the United States Census Bureau, the year 2030 will present a demographic transition for the population when all baby boomers will be older than 65 and nearly 20% of Americans will be retirement age (1). The country’s 65-and-older population is projected to double from 2016 to 2060, increasing to nearly 25% of the projected population, and the number of people 85 years and older is projected to double by 2035 and triple by 2060 (1). These demographic shifts are expected to bring with them a similar increase in cancer diagnoses, as cancer occurs most commonly in older adults (2). These impending changes will likely exert substantial stress on the health care system and portend the need to optimize treatments in the senior adult population. To date, senior adult patients have been largely under-represented in cancer clinical trials, making them vulnerable to treatment disparities (2–4). Additionally, aging confers physiological changes in nearly every body system, such as decreased cardiac output, immune system depression, impaired wound healing, and vascular changes, that increase the risk of morbidities associated with standard cancer treatments (5). Therefore, physicians will likely encounter increasing challenges in treating senior adult cancer patients whose goals of care may influence them to pursue treatment plans that deviate from accepted standards of care (6).

According to the Center for Disease Control and Prevention, approximately half of head and neck cancer cases are diagnosed in patients older than 65 years, with a large proportion of those cases being diagnosed in patients older than 70 years (7). For locally advanced HNSCC, the majority of patients are treated with combined treatment modalities, with approximately 40% of patients receiving primary surgery + adjuvant radiation +/- chemotherapy versus 50% receiving primary chemoradiotherapy (8). Moreover, the National Comprehensive Cancer Network (NCCN) provides evidence-based treatment guidelines for various cancer types based on tumor stage and characteristics. Guideline adherence is generally associated with improved survival outcomes, while deviance is associated with complications or recurrence (9–14). The NCCN guidelines recommend primary surgical resection with adjuvant radiation +/- chemotherapy based on final pathology for locoregionally advanced HNSCC (15). All treatments come with expected and possible side effects that effect quality of life. These sequalae are anticipated and tolerated when there is a clear oncologic advantage. Radiotherapy (RT) plays a key-role in curative-intent treatments of both early and late-stage disease but comes with the risk of early and late toxicities (16–18). When the sequela of treatment are compounded with the physiological changes of normal aging, clinicians must reevaluate the risks versus benefits of the treatment course in senior adult patients, which has been well-characterized in oncologic literature as shared decision making (6, 19–23). The current study used the National Cancer Database (NCDB) to analyze the survival benefits associated with adjuvant radiotherapy for senior adult patients with locally advanced HNSCC who undergo primary surgical resection.

## Materials and methods

**Figure 1:**
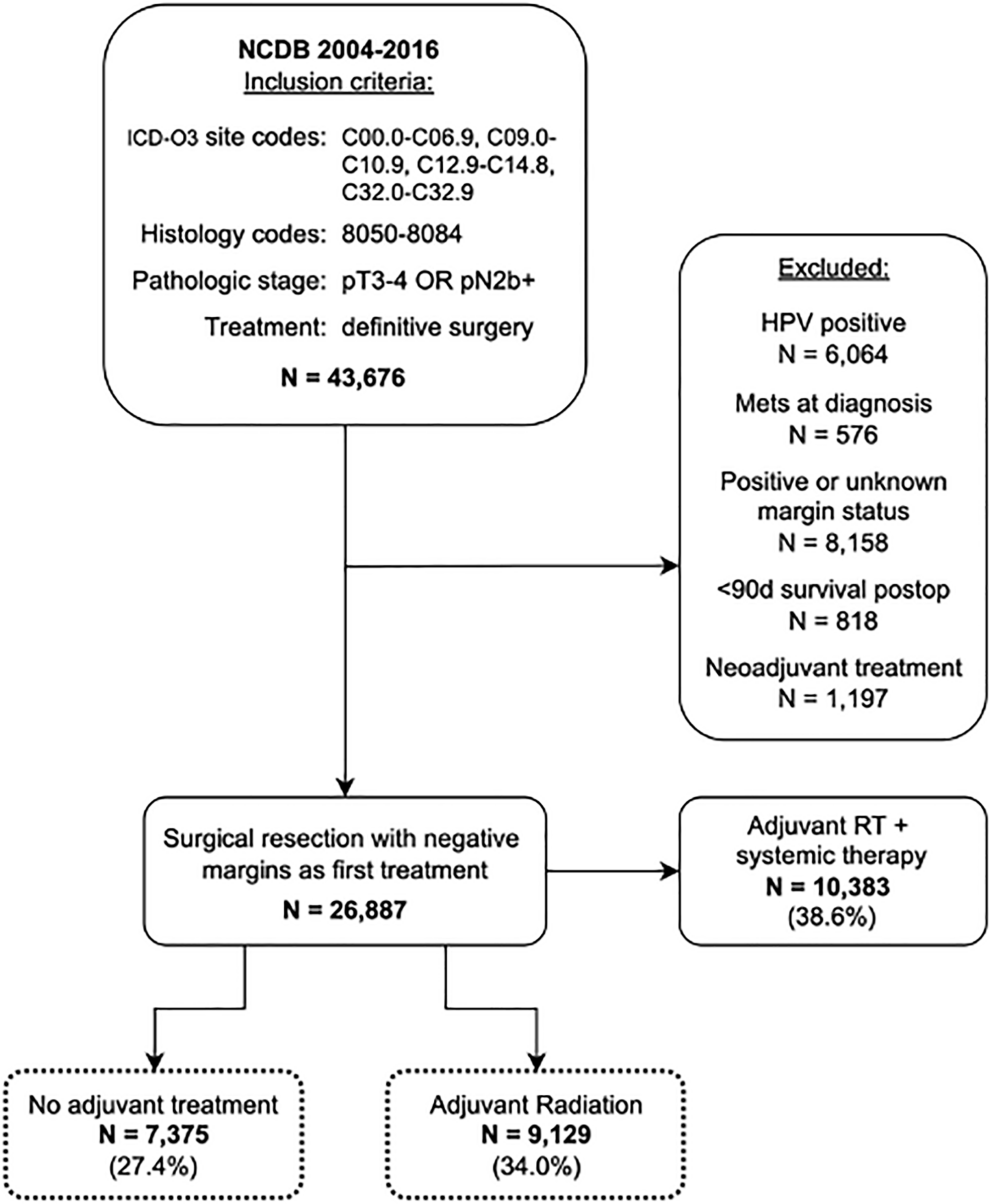
Cohort selection from NCDB.

The National Cancer Database (NCDB) was queried for all cases of HNSCC from 2004-2016 using the International Classification of Disease for Oncology-3 (ICD-O3) topographical and morphological codes. Topographical codes included C00.0-C06.9, C09.0-10.9, C12.9-C14.8, and C32.0-32.9. Additional details on primary sites and ICD-O3 codes can be found in **Supplemental Table 1**. Squamous cell carcinoma histology was identified using ICD-O3 morphological codes 8050-8084. Patients were included if they were diagnosed with advanced locoregional disease, defined as pT3/T4 and/or N2b or greater without distant metastasis, as their first or only cancer. All included patients underwent definitive surgical resection with or without adjuvant RT. Exclusion criteria included HPV-positive disease, positive surgical margins, death within 90 days of surgical resection, administration of neoadjuvant treatment, treatment with adjuvant chemo- or immunotherapy, treatment with adjuvant RT with systemic therapy, or any missing variables pertaining to the American Joint Committee of Cancer (AJCC) clinical or pathological stage (**Figure 1**). Treatment with neoadjuvant therapy was defined as administration of chemotherapy or RT ≤90 days before surgery. Adjuvant RT was defined as a course of external beam radiation initiated after surgery as part of the planned first course of treatment. As per institutional guidelines, analysis of deidentified data does not require institutional review board approval. The final cohort included 16,504 patients.

Patients were grouped into adult (age <80 years) and senior adult (age ≥80 years) cohorts. The age 80 years was greater than one standard deviation from the mean population age (mean = 63.8 years, SD = 12.0) and was therefore chosen as the cutoff age for our senior adult cohort. Within each cohort, patients were divided into treatment groups based on whether they received adjuvant RT. Overall survival (OS) was compared between treatment groups within adult and senior adult cohorts and was assessed at 1, 3, and 5 years after surgery using Kaplan Meier estimates. The log-rank tests were used to assess significance. Cox proportional hazards analysis was used to further examine the impact of adjuvant RT on OS at these same time points while accounting for differences in cohort characteristics identified in **Table 1**. A Cox regression model was built with fixed covariates including patient age, sex, race, Charlson-Deyo comorbidity score, primary site, AJCC pathologic stage, and treatment type. Hazard ratios were assessed at 1, 3, and 5 year time points. The analysis was performed within IBM SPSS Statistics for Macintosh, version 27 (IBM Corp., Armonk, N.Y., USA) (24). Statistical significance was defined as a p-value<0.05.

**Table 1:** Patient and tumor characteristics by age group and treatment type. additional analysis of cohort demographics can be found in **Table 2** and **Supplemental Tables 1**, **2**.

	Adults (Age <80)		Senior Adults (Age 80+)	
	Surgery AloneN = 6,250	Surgery + Adj RTN = 8,389	Surgery AloneN = 1,125	Surgery + Adj RTN = 750
Age (mean [SD])	60.7 [10.1]	62.1 [10.1]	83.4 [2.9]	84.4 [3.3]
Sex				
Male	71.6%	71.4%	47.3%	49.5%
Female	28.4%	28.6%	52.7%	50.0%
Race				
White	84.7%	83.6%	90.0%	90.1%
Black	11.0%	11.5%	5.1%	4.7%
Other/unknown	4.3%	4.9%	4.0%	5.1%
Charlson-Deyo score				
0	67.8%	71.9%	64.7%	72.4%
1	22.6%	20.8%	22.8%	19.9%
2	6.9%	5.1%	8.2%	5.8%
3+	2.7%	2.3%	4.4%	1.9%
Primary site				
Oral Cavity	48.1%	53.5%	75.4%	79.9%
Oropharynx	11.7%	13.7%	5.9%	6.4%
Hypopharynx	5.1%	3.3%	3.0%	2.3%
Larynx	35.0%	29.5%	15.7%	11.5%
AJCC pathologic stage				
3	30.2%	18.0%	24.5%	11.4%
4	69.8%	82.0%	75.5%	88.6%

## Results

### Cohort characteristics

A total of 16,504 patients met inclusion criteria. Characteristics of each cohort are outlined in **Table 1**. The mean age of the total population was 63.8 years (SD 12.0 years). The adult cohort included 14,639 (88.7%) patients with a mean age of 61.3 years (SD 10.1 years). The senior adult cohort included 1,865 (11.3%) patients with a mean age of 84.0 years (SD 3.21 years). In both cohorts, patients undergoing adjuvant RT were identified for comparison to those treated with surgery alone. Adjuvant RT was utilized in a significantly higher proportion of patients <80 years old relative to senior adults (**Table 2**). Additionally, prior to exclusion of all patients receiving adjuvant systemic therapy, adjuvant RT was utilized in 72.6% of patients overall.

**Table 2:** Treatment types and radiation dose by age group.

	Adults (Age <80) N = 14,639	Senior Adults (Age 80+) N = 1,865	p
Treatment			<0.001
Surgery alone	42.7%	60.3%	
Surgery + adj RT	57.3%	39.7%	
Adequate adj RT dose*			0.002
Yes (≥60 Gray)	87.8%	83.7%	
No (<60 Gray)	12.2%	16.3%	

*Valid percent for patients with available data: Age <80: N=7,484; Age 80+: N=667.

### Kaplan Meier survival analysis

**Figure 2:**
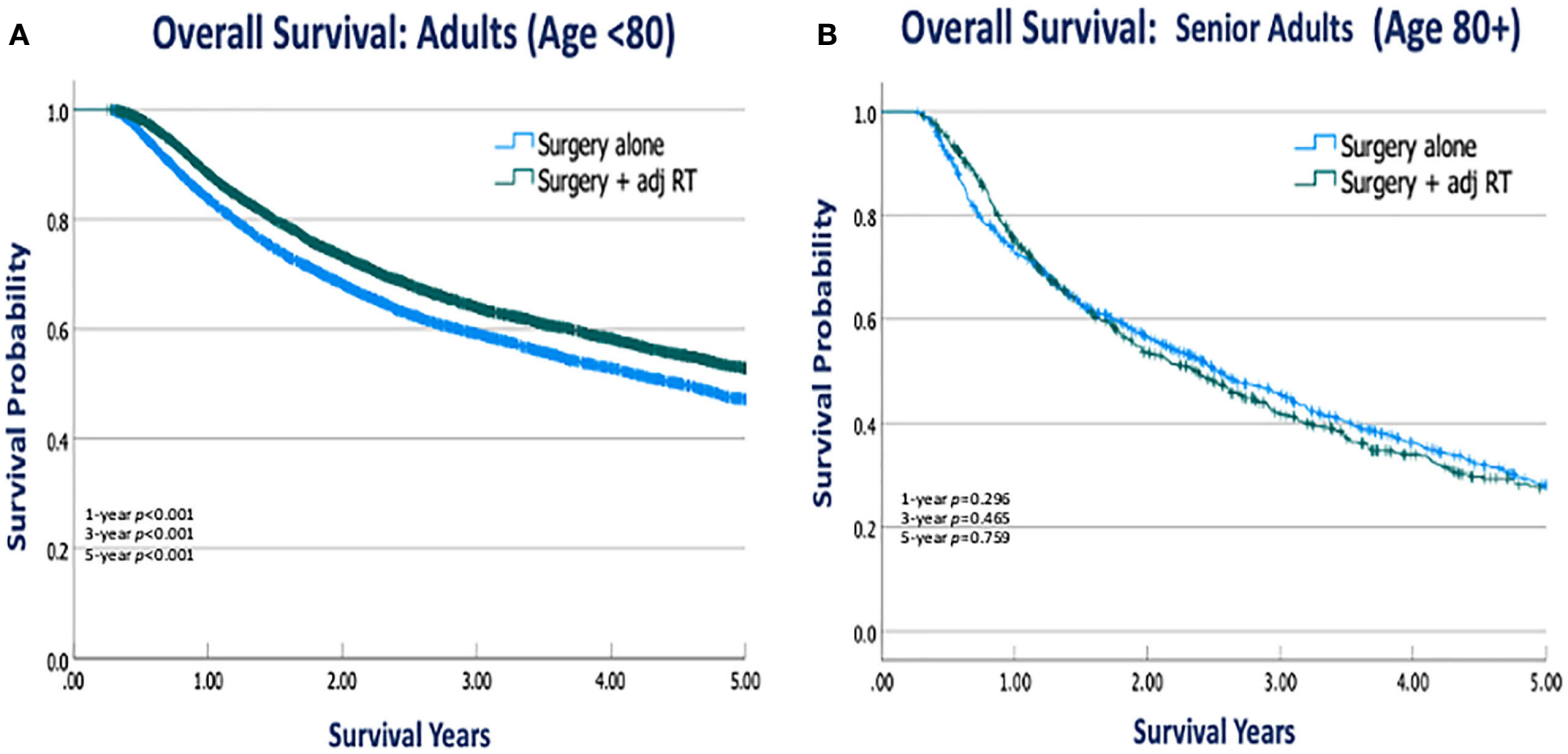
Kaplan Meier OS curves stratified by treatment group within adult and senior adult patient cohorts. **(A)** OS for Adults (Ages<80) stratified by treatment type **(B)** OS for Senior Adults (Age 80+) stratified by treatment type.

Within each age group, overall survival was compared between patients who underwent surgery alone versus those receiving adjuvant RT. Kaplan Meier OS curves were stratified by treatment groups within each age cohort (**Figure 2**). OS did not differ significantly between treatment groups in senior adults, whereas those ages <80 years demonstrated a loss of OS when they did not receive adjuvant RT. When assessed at specific time points, Kaplan Meier estimates of OS showed that adjuvant RT was associated with significantly higher OS in the adult cohort at 1-year (88.4% vs. 83.8%, p<0.001), 3-years (64.0% vs. 59.2%, p<0.001), and 5-years (52.8% vs. 47.2%, p<0.001) post-op compared to surgery alone. In the senior adult cohort, adjuvant RT was not associated with significantly different OS at 1-year (74.8% vs. 73.4%, p=0.296), 3-years (45.8% vs. 41.8%, p=0.465), and 5-years (28.2 vs. 27.7%, p=0.759) compared to surgery alone (**Table 3**).

**Table 3:** Kaplan Meier estimates of OS by age cohort and treatment group.

Adults (Age<80) N=14,639 (88.7%)			
	Surgery Alone N=6,250 (42.7%)	Surgery + Adj RT N=8,389 (57.3%)	p
1-year OS	83.80%	88.40%	<0.001
3-year OS	59.20%	64.00%	<0.001
5-year OS	47.20%	52.80%	<0.001
**Senior Adults**			
**(Age 80+)**			
**N=1,865 (11.3%)**			
	**Surgery Alone N=1,125 (60.3%)**	**Surgery + Adj RT N=740 (39.7%)**	**p**
1-year OS	73.40%	74.80%	0.296
3-year OS	41.80%	45.80%	0.465
5-year OS	27.70%	28.20%	0.759

### Corrected survival analysis

To account for significant differences in patient and tumor characteristics between treatment groups, Cox proportional hazards model was used to examine the impact of adjuvant RT on overall survival by analyzing the OS hazard ratio (HR) between treatment groups within the age cohorts. This was performed separately for the senior adult and adult cohorts at 1-, 3-, and 5-years post-op. All variables found to be significantly different between treatment groups in **Table 1** were included in the regression model. Variables included age, sex, race, Charlson-Deyo comorbidity score, primary site, and AJCC pathologic stage. Surgery plus adjuvant therapy was used as the reference value. In the adult cohort, surgery alone remained a significant predictor of mortality risk at 1-year (HR 1.48, 95% CI 1.35-1.64, p<0.001), 3-years (HR 1.25, 95% CI 1.18-1.33, p<0.001), and 5-years (HR 1.23, 95% CI 1.17-1.30, p<0.001) post-op when accounting for other patient characteristics. In the senior adult cohort, adjuvant RT did not significantly impact mortality risk at 1-year (HR 1.11, 95% CI 0.90-1.36, p=0.316), 3-years (HR 0.94, 95% CI 0.81-1.08, p=0.423), or 5-years (HR 0.95, 95% CI 0.83-1.08, p=0.476) post-op relative to surgery alone (**Table 4**). Hazard ratios of OS did not significantly differ between treatment groups in senior adults, whereas the omission of adjuvant RT in those ages <80 years significantly increased mortality risk. The Cox proportional hazards model for 3-year mortality is shown in **Table 5**. Results of the 1-year and 5-year models can be found in **Supplemental Tables 2** and **3**, respectively.

**Table 4:** Cox multivariate regression analysis of OS HR by treatment type in each age cohort.

Adults (Age<80)				
	Treatment	HR	95% CI	p
**1-year mortality**	Surgery + adj RT	1	–	<0.001
	Surgery alone	1.48	1.35-1.64	
**3-year mortality**	Surgery + adj RT	1	–	<0.001
	Surgery alone	1.25	1.18-1.33
**5-year mortality**	Surgery + adj RT	1	–	<0.001
	Surgery alone	1.23	1.17-1.30
**Senior Adults**				
**(Age 80+)**				
	**Treatment**	**HR**	**95% CI**	**p**
**1-year mortality**	Surgery + adj RT	1	–	0.316
	Surgery alone	1.11	0.90-1.36	
**3-year mortality**	Surgery + adj RT	1	–	0.423
	Surgery alone	0.94	0.81-1.08	
**5-year mortality**	Surgery + adj RT	1	–	0.476
	Surgery alone	0.95	0.83-1.08	

**Table 5:** Cox proportional hazards model for 3-year mortality.

	Adults (Age <80)			Senior Adults (Age 80+)		
	HR	95% CI	p	HR	95% CI	p
Age (mean [SD])	1.017	(1.014 – 1.020)	<0.001	1.037	(1.014 - 1.060)	<0.001
Charlson-Deyo score						
0	1.0	-		1.0	-	
1	1.133	(1.054 – 1.218)	<0.001	1.064	(0.900 – 1.259)	0.468
2	1.493	(1.332 – 1.675)	<0.001	1.289	(0.989 – 1.679)	0.060
3+	1.692	(1.415 – 2.023)	<0.001	1.549	(1.083 – 2.216)	0.017
Primary site						
Oral Cavity	1.0	-		1.0	-	
Oropharynx	0.708	(0.640 – 0.783)	<0.001	0.935	(0.700 – 1.249)	0.649
Hypopharynx	1.297	(1.136 – 1.480)	<0.001	1.795	(1.254 – 2.579)	0.001
Larynx	0.839	(0.782 – 0.899)	<0.001	0.841	(0.682 – 1.037)	0.105
AJCC pathologic stage						
3	1.0	-		1.0	-	
4	1.519	(1.405 – 1.643)	<0.001	1.208	(1.006 – 1.451)	0.043
Treatment type						
Surgery alone	1.25	(1.18 – 1.33)	<0.001	0.94	(0.81 – 1.08)	0.336
Surgery + Adj RT	1.0	-		1.0	-	

## Discussion

In this retrospective analysis of the NCDB, we found no significant difference in OS in senior adult patients with locally advanced HNSCC who underwent primary surgical resection with or without adjuvant RT, while adjuvant RT was associated with a significant increase in OS in adult patients. A significant difference in adjuvant RT utilization between senior adults (39.7%) and adults (57.3%) (p<0.001) likely represents a de-escalation of treatment as patients age (25). In senior adults, adjuvant RT did not significantly impact the adjusted mortality risk relative to surgery alone when accounting for differences in patient characteristics, primary site, and AJCC pathologic stage. In adults, treatment with surgery alone remained a significant predictor of mortality when accounting for the same variables.

The literature is mixed regarding which treatment modalities are best for senior adult patients with HNSCC. Roden et al. (2019) found no significant differences in OS in 159 older adult HNSCC patients (≥80 years) who received NCCN guideline-directed adjuvant therapy compared to those who deviated from the recommendations for adjuvant treatment (13). Moreover, Haehl et al. (2020) compared outcomes in 246 older adult HNSCC patients (age ≥75 years) who underwent definitive or adjuvant treatment with chemoradiation (CRT) or RT and found no significant differences in OS but noted prevalent treatment-associated toxicities (26). On the contrary, multiple other studies support that senior adults should be treated with conventional protocols if their baseline status allows (27–29). The ambivalence in recent literature could suggest an age-based transition point in outcomes where the risks associated with aggressive curative-intent therapy begin to mitigate its benefits.

While the benefits of adjuvant therapies are well-studied, so are the adverse effects. (13, 30, 31) Several studies report that physiologic changes contribute to age as a risk factor for complications from recommended therapy for advanced HNSCC, including aspiration pneumonia, dysphagia, mucositis, and dermatitis (5, 26, 27). Moreover, multiple unique studies of the SEER Medicare-linked database found that the development of treatment-associated morbidities was higher in older adults who received adjuvant RT compared to those who received surgery alone (32–34). However, the risk of adverse effects varies with tumor location and may influence shared decision-making regarding adjuvant therapy based on primary site. In addition to physiologic changes, several studies recognize increasing comorbidity burden as a contributor to increased susceptibility to treatment toxicity in older adult HNSCC patients, and geriatric assessment tools for treatment tolerability are yet to be optimized (35–37). Despite ambiguity about the role adjuvant therapy should play in senior adult patients, these data should encourage clinicians and patients to discuss the risks and benefits of the current standard of care based on patient goals, baseline functional status, and tumor primary site.

Our adjuvant RT treatment cohorts included patients who may have received partial courses to avoid introducing selection bias favoring healthier patients who are able complete adjuvant RT. Multiple studies suggest that early termination of adjuvant RT or prolonged interruptions have negative impacts on survival outcomes in older HNSCC patients, though advanced age itself is a debatable predictor of the likelihood of treatment completion (26, 37–40). This could represent an important distinction in the management of these patients by underscoring the importance of adjuvant RT course completion to receive survival benefits. Taken together, it is reasonable to recognize early termination or discontinuity of adjuvant RT as a risk factor for poorer outcomes and acknowledge that advanced age may pose increased challenges in treatment completion, be that from increased susceptibility to toxicity, increased comorbidity burden, or social factors limiting access to treatment.

One interpretation of our findings in the context of recent literature is that perhaps the survival benefit that adjuvant RT confers is limited to completed treatment courses, and interrupted or prematurely terminated adjuvant RT courses can cause adverse effects with negligible benefit. Our data captured both complete and incomplete treatment courses and concluded that adjuvant RT neither improves nor decreases OS compared to surgical resection alone, perhaps representing an average of the results seen in the aforementioned studies. Adjuvant RT should not be offered or withheld based on age, but great care should be taken to ensure that senior adult patients complete the entire course with minimal interruptions to reap survival benefits. If there is uncertainty regarding treatment tolerability or appropriate support to complete a full course, then it may be advisable to forego adjuvant RT to avoid survival curtailment from interruption/incompletion. Perhaps an “all or nothing” approach to adjuvant RT in senior adults can maximize OS for fit patients and minimize risks for those with greater frailty or fewer supportive resources.

Ultimately, there is a need for more comprehensive prospective data and clinical trials for senior adult HNSCC patients, as much of the current understanding is limited to large database studies or institutional retrospective analysis. We acknowledge several limitations to this study. The data contained in the NCDB do not contain the entire United States population and may be subject to biases regarding geography, urban versus rural reporting, and selection bias favoring patients who have access to a Commission on Cancer-approved hospital. The data’s reliability also depends on submission accuracy from participating hospitals. Additionally, analysis is limited to OS as the NCDB does not contain disease-specific mortality information, and in-depth analysis of specific treatment regimens is limited by the lack of specific radiation protocols and rationale for treatment selection included in the NCDB. Further research is needed to explore differences in disease-specific survival, disease-free survival, and quality of life measures to guide shared-decision making in this population. Prospective studies or clinical trials involving this population would provide a more comprehensive understanding of optimal treatments.

## Conclusion

This study analyzed of the effects of adjuvant RT on OS in senior adult patients with locally advanced HNSCC using 16,504 cases from the NCDB. We found that the addition of adjuvant RT to definitive surgical treatment in senior adult patients (age ≥80 years) may not provide a similar OS benefit to that observed in younger patients.

## Previous presentation statement

This article was presented at the ASTRO Multidisciplinary Head and Neck Cancers Conference, February 24-26, 2022, in Phoenix, AZ, and the Pennsylvania Academy of Otolaryngology- Head and Neck Surgery Annual Scientific Conference, June 17-18, 2022, in Hershey, PA.

## Data availability statement

The data analyzed in this study is subject to the following licenses/restrictions: The National Cancer Database Participant User Files are available through an application process. Requests to access these datasets should be directed to https://www.facs.org/quality-programs/cancer-programs/national-cancer-database/puf/.

## Ethics statement

Ethical review and approval was not required for the study on human participants in accordance with the local legislation and institutional requirements. Written informed consent for participation was not required for this study in accordance with the national legislation and the institutional requirements.

## Author contributions

JB: study design, data collection, analysis, and manuscript writing/editing; MC: study design, data collection, analysis, and manuscript writing/editing; VB-A: manuscript writing/editing. AL: study design and manuscript writing/editing. All authors contributed to the article and approved the submitted version.

## Conflict of interest

The authors declare that the research was conducted in the absence of any commercial or financial relationships that could be construed as a potential conflict of interest.

## Publisher’s note

All claims expressed in this article are solely those of the authors and do not necessarily represent those of their affiliated organizations, or those of the publisher, the editors and the reviewers. Any product that may be evaluated in this article, or claim that may be made by its manufacturer, is not guaranteed or endorsed by the publisher.
