# Supplementary material for: Impact of adjuvant radiation therapy after definitive surgery in senior adults >80 years old with advanced head and neck squamous cell carcinoma on overall survival
Source: Front Oncol. 2022 Sep 30;12:973245. doi: 10.3389/fonc.2022.973245 (PMC9562704; doi:10.3389/fonc.2022.973245)
Supplement: Supplementary file 1 [file DataSheet_1.pdf]

Supplemental Table 1. HNSCC primary sites and corresponding ICD-O3 codes used for analysis.

Primary Site	ICD-O3 Code
Mucosa of lip	C00.3-C00.5
Base of tongue	C01.9
Tongue	C02.0-C2.3, C02.8, C02.9
Tonsil- lingual, palatine	C02.4, C09.0, C09.1, C09.8, C09.9
Gingiva	C03.0, C03.1, C03.9
Floor of mouth	C04.0, C04.1, C04.8, C04.9
Palate- hard, soft	C05.0, C05.1, C05.8, C05.9
Uvula	C05.2
Oral cavity	C06.0-C06.2, C06.8, C06.9
Vallecula	C10.0
Epiglottis	C10.1
Oropharynx	C10.2-C10.4, C10.8, C10.9
Pyriform sinus	C12.9
Hypopharynx	C13.0-C13.2, C13.8, C13.9
Pharynx	C14.0, C14.2, C14.8
Larynx	C32.0-C32.3, C32.8, C32.9

Supplemental Table 2. Cox proportional hazards model for 1-year mortality.

	Age <80			Age 80+		
	HR	95% CI	p	HR	95% CI	p
Age (mean [SD])	1.022	(1.017 – 1.027)	<0.001	1.057	(1.025 – 1.090)	<0.001
Charlson-Deyo score						
0	1.0 (ref)	-		1.0 (ref)	-	
1	1.205	(1.017 – 1.027)	0.002	0.947	(0.736-1.219)	0.673
2	1.538	(1.284 – 1.841)	<0.001	1.520	(1.066 – 2.168)	0.021
3+	1.882	(1.438 – 2.464)	<0.001	1.447	(0.858 – 2.439)	0.166
Primary site						
Oral Cavity	1.0 (ref)	-		1.0 (ref)	-	
Oropharynx	0.739	(0.625 – 0.874)	<0.001	1.034	(0.686 – 1.558)	0.875
Hypopharynx	1.243	(1.003 – 1.539)	0.047	1.790	(1.107 – 2.893)	0.017
Larynx	0.840	(0.750 – 0.941)	0.003	0.682	(0,488 – 0.953)	0.025
AJCC Pathologic Stage						
3	1.0 (ref)	-		1.0 (ref)	-	
4	1.543	(0.358 – 1.754)	<0.001	1.357	(1.034 – 1.781)	0.028
Treatment						
Surgery alone	1.0 (ref)	-		1.0 (ref)	-	
Surgery + adj RT	0.671	(0.609 – 0.740)	<0.001	0.895	(0.726 – 1.103)	0.297

Supplemental Table 3. Cox proportional hazards model for 5-year mortality.

	Age <80			Age 80+		
	HR	95% CI	p	HR	95% CI	p
Age (mean [SD])	1.018	(1.015 – 1.021)	<0.001	1.048	(1.027 – 1.070)	<0.001
Charlson-Deyo score						
0	1.0 (ref)	-		1.0 (ref)	-	
1	1.148	(1.075 – 1.227)	<0.001	1.029	(0.880 – 1.202)	0.722
2	1.439	(1.292 – 1.602)	<0.001	1.256	(0.979 – 1.612)	0.073
3+	1.799	(1.524 – 2.121)	<0.001	1.487	(1.047 – 2.114)	0.027
Primary site						
Oral Cavity	1.0 (ref)	-		1.0 (ref)	-	
Oropharynx	0.709	(0.646 – 0.778)	<0.001	1.039	(0.798 – 1.352)	0.775
Hypopharynx	1.370	(1.214 – 1.574)	<0.001	1.795	(1.274 – 2.530)	<0.001
Larynx	0.891	(0.837 – 0.949)	<0.001	1.016	(0.846 – 1.220)	0.864
AJCC Pathologic Stage						
3	1.0 (ref)	-		1.0 (ref)	-	
4	1.018	(1.015 – 1.021)	<0.001	1.205	(1.020 – 1.422)	0.028
Treatment						
Surgery alone	1.0 (ref)	-		1.0 (ref)	-	
Surgery + adj RT	0.814	(0.770 – 0.861)	<0.001	1.057	(0.925 – 1.208)	0.416
